# Potential drug-drug interactions and their risk factors in pediatric patients admitted to the emergency department of a tertiary care hospital in Mexico

**DOI:** 10.1371/journal.pone.0190882

**Published:** 2018-01-05

**Authors:** Olga Morales-Ríos, Luis Jasso-Gutiérrez, Alfonso Reyes-López, Juan Garduño-Espinosa, Onofre Muñoz-Hernández

**Affiliations:** 1 Departamento de Evaluación y Análisis de Medicamentos, Hospital Infantil de México Federico Gómez, Ciudad de México, México; 2 Centro de Estudios Económicos y Sociales en Salud, Hospital Infantil de México Federico Gómez, Ciudad de México, México; 3 Dirección de Investigación, Hospital Infantil de México Federico Gómez, Ciudad de México, México; 4 Comisión Nacional de Arbitraje Médico, Ciudad de México, México; Università degli Studi di Milano, ITALY

## Abstract

**Background:**

Drug-drug interactions (DDIs) detected in a patient may not be clinically apparent (potential DDIs), and when they occur, they produce adverse drug reactions (ADRs), toxicity or loss of treatment efficacy. In pediatrics, there are only few publications assessing potential DDIs and their risk factors. There are no studies in children admitted to emergency departments (ED). The present study estimates the prevalence and describes the characteristics of potential DDIs in patients admitted to an ED from a tertiary care hospital in Mexico; in addition, potential DDI-associated risk factors are investigated.

**Methods:**

A secondary analysis of data from 915 patients admitted to the ED of the Hospital Infantil de México “Federico Gómez” was conducted. The Medscape Drug Interaction Checker software was used to identify potential DDIs. The results are expressed as number of cases (%), means (95% CI) and medians (25-75^th^ percentiles). Count data regressions for number of total and severity-stratified potential DDIs were performed adjusting for patient characteristics, number of administered drugs, days of stay, presence of ADRs and diagnoses.

**Results:**

The prevalence of potential DDIs was 61%, with a median of 4 (2–8). A proportion of 0.2% of potential DDIs was “Contraindicated”, 7.5% were classified as “Serious”, 62.8% as “Significant” and 29.5% as “Minor”. Female gender, age, days of stay, number of administered drugs and diagnoses of Neoplasms (C00-D48), Congenital malformations (Q00-Q99), Diseases of the Blood, Blood-forming Organs and Immunity (D50-D89) and Diseases of the nervous system (G00-G99) were significantly associated with potential DDIs.

**Conclusion:**

The prevalence of potential DDIs in the ED is high, and strategies should therefore be established to monitor patients’ safety during their stay, in addition to conducting investigations to estimate the real harm potential DDIs inflict on patients.

## Introduction

Drugs are a tool in medical practice and constitute and important advance in pharmaceutical sciences. However, the prescription of multiple drugs to a patient can favor the presence of drug-drug interactions (DDIs), which can be identified when the pharmacological or clinical response to the administration of a combination of two drugs is different than expected based on both drugs’ known effects when individually prescribed [1]. The “Potential Drug-Drug Interaction” (potential DDI) concept refers to the possibility a drug has to alter the effects of another when both are simultaneously administered [2]. In medical practice, it is quite common using drug combinations with the capability to interact, and although not all DDIs detected in a patient may occur (potential DDIs), their identification is relevant since they can increase the risk for adverse drug reactions (ADRs), toxicity or loss of treatment efficacy, which in addition to adverse consequences for patients, can increase days of hospital stay and costs [3–6]. Children can be more vulnerable to the occurrence of potential DDIs than adults because: **a)** hospitalized children can be administered more than 25 drugs during their stay [7], **b)** they can react differently to drug administration than adults, which is explained by changes in absorption, distribution, metabolism and excretion [8], and **c)** unlicensed and off-label prescription of drugs [9].

In the pediatric population, the prevalence of potential DDIs ranges from 3.8% to 75% [10–16]. With regard to risk factors associated with potential DDIs, the risk in hospitalized children was found to increase with patient age, average number of prescriptions per visit, number of visits per year, some diagnoses (epilepsy, leukemia, rheumatoid arthritis) and groups of drugs (antiepileptic, anti-neoplastic, systemic antifungal and immunosuppressant drugs, as well as those used for respiratory tract obstructive conditions) [10]. In the case of onco-hematological pediatric patients, the risk for a potential DDI according to Micromedex [14] has been reported to increase with the male gender, with an underlying diagnosis of hematological diseases and with an elevated number of non-antineoplastic drugs prescribed, whereas according to Drug Interaction Facts, the risk for a potential DDI is significantly increased with the male gender, emergency hospital admission and total number of prescribed drugs [14]. Another study conducted in patients of a pediatric intensive care unit (PICU) reports that potential DDIs are associated with Caucasian ethnicity, some diagnoses (neoplasms, diseases of the circulatory system, congenital anomalies and diseases of the nervous system), presence of complex chronic conditions, increase in daily average exposure to drugs and increase in the number of days of PICU stay [16]. In the case of adult patients, different studies have been published reporting potential DDIs frequency, characteristics and risk factors [1,17–18], with two of these studies having been conducted in Mexico [1,18]. Among the studies that have reported potential DDIs frequency and characteristics, as well as their associated risk factors in the pediatric population, there wasn’t any identified that was carried out in the setting of an Emergency Department (ED), which is highly important, since especially children admitted to the ED have severe, life-threatening conditions, and this is a situation that makes them more susceptible to multiple drug administration, complex treatment regimens, prolonged ED stays and care by different specialist physicians for multiple consultations [19–20]. In addition, no publications were identified about studies conducted in Mexican children assessing potential DDIs, as well as their associated risk factors.

### Aim of the study

In view of all this, the purposes of this work were: a) to estimate the prevalence and describe the characteristics of potential DDIs in pediatric patients admitted to the ED in a tertiary care pediatric hospital of Mexico and b) to evaluate the risk factors associated with the presence of potential DDIs.

## Methods

### Study design

The present study is a secondary data analysis from a comprehensive intervention for adverse drug reactions identification and reporting in a pediatric ED [21]. The period of study was from March 2012 to June 2013. Since the patients were studied at a point in time, we can claim this is a cross-sectional study.

### Study population and setting

The study was conducted at the ED of the Hospital Infantil de México Federico Gómez (HIMFG), which is a national pediatric health institute in México. The study population of the intervention study included 1,179 inpatients, out of which only 915 were selected for this analysis because they were treated with two or more drugs.

### Procedure for potential drug-drug interactions identification

During the intervention study [21], one of the authors of this work (OMR) collected the information from the medical record of each patient who was admitted to the ED. The collected data included: age, gender, weight, height, diagnoses, date of admission and discharge, presence of adverse drug reactions (ADRs), and medications received during the ED stay (drug name, dose, administration route and date). When a medication contained two or more pharmacological compounds, each drug was individually considered in the analysis. Each patient’s medication list was screened using Medscape Drug Interaction Checker [22], which is freely accessible software. All identified potential DDIs were recorded and graded according to their level of severity, and a description was given on their mechanism of action and adverse consequences, as well as recommendations. By definition, a potential DDI categorized as “Contraindicated” is a combination of drugs that never be used because there is high risk of dangerous interaction. The “Serious-Use alternative” category indicates that there is potential for serious interaction, and regular monitoring by the treating physician is required or alternate medication may be needed. The “Significant-Monitor Closely” category refers to the possibility of significant interaction (monitoring by treating physician is likely required). A “Minor” categorization means that interaction is unlikely, minor, or non-significant.

### Data analyses

The prevalence of potential DDIs was defined as the number of patients with any potential DDI divided by the total number of patients that received two or more drugs in the study period and multiplied by 100. Potential DDIs total number was defined as the number of potential DDIs detected by means of Medscape Drug Interaction Checker [22]. The percentage of patients with at least one Contraindicated, Serious, Significant and Minor potential DDIs was defined as the number with at least one of these potential DDIs divided by the total number of patients who had potential DDIs and then multiplied by 100. For descriptive purposes, patients were classified in 4 categories according to the body mass index Z-score (normal weight, obesity, overweight and underweight) [23] and in 3 categories according to age (infants, children and adolescents) [24]. The ICD-10 was used for all diagnoses [25]. The number of medications was defined as the total number of drugs administered to the patient in the ED.

#### Descriptive data analysis

The univariate data analysis included central tendency and dispersion measures estimation for quantitative variables, and relative frequencies (95% CIs) for qualitative variables. The bivariate data analysis included the comparison between those patients with and without at least one potential DDI. For the age, height, weight, number of medications and number of days of ED stay variables, the Kolmogorov-Smirnov nonparametric test was selected because these variables did not show a normal distribution based on the Shapiro-Francia test. For categorical variables (age group, gender, body mass index category according to Z-score, and diagnosis), Fisher's exact test was used.

#### Multivariable regression analysis

To assess risk factors associated with the presence of potential DDIs, several count data regression models were adjusted, where the dependent variable was the number of potential DDIs (total and stratified by severity).

#### Model selection

The highly right-skewed distribution of the counts (total and severity-stratified potential DDIs) recorded from our patient sample is shown in S1 Fig, which is indicative for using Zero-inflated count regression models (the Zero-inflated Poisson model (ZIP) or the Zero-inflated negative binomial model (ZINB)) to evaluate the factors associated with that distribution. The ZIP model assumes a Poisson distribution of the response variable, where the mean is equal to the variance; i.e., it accepts the existence of equidispersion. However, when the above is not possible, the ZINB model has the flexibility for modeling data with overdispersion. Based on the above, the first strategy to select the best model was to assess the overdispersion parameter (α) with the Likelihood ratio (LR) test, and when it was statistically significant, it allowed for the presence of overdispersion to be concluded and, as a consequence, the ZINB model was used. The second confirmatory strategy was to calculate the Akaike (AIC) and Bayesian (BIC) information criteria, which, when compared between the ZIP and ZINB models, allowed for the model that obtained the lowest value in both statistics to be chosen. Zero-inflated count models respond to the failure of the Poisson regression model to account for dispersion and excess zeros by changing the mean structure to allow zeros to be generated by two distinct processes. The zero-inflated model assumes that there are two latent (i.e., unobserved) groups. An individual in the Always-0 Group has an outcome of 0 with a probability of 1, while an individual in the Not Always-0 Group might have a zero count, but there is a nonzero probability that she has a positive count. This process is developed in three steps: Step 1) Model membership into the latent groups; Step 2) Model counts for those in the Not Always-0 Group; and Step 3) Compute observed probabilities as a mixture of the probabilities for the two groups [26]. If we define membership in the *Always-0 Group as membership in* Group A, and let A = 1 if someone is in Group A, else A = 0, then group membership is a binary outcome that can be modeled using a logit or probit model,

$$\psi_{i}=\mathrm{Pr}\left(A_{i}=1|z_{i}\right)=F(z_{i}\gamma )$$

where *ψ*_*i*_ is the probability of being in Group A for individual *i*. The **z**-variables are referred to as inflation variables since they serve to inflate the number of 0s. To illustrate the previous equation, assume that two variables affect the probability of an individual being in Group A and that we model this with a logit equation:

$$\psi_{i}=\frac{exp(\gamma_{0}+\gamma_{1}z_{1}+\gamma_{2}z_{2})}{1+exp(\gamma_{0}+\gamma_{1}z_{1}+\gamma_{2}z_{2})}$$

If we had an observed variable indicating group membership, this would be a standard, binary regression model. But, since group membership is a latent variable, we do not know whether an individual is in Group A or the *Not Always-0 Group* [26].

#### Covariates included in the models

The covariates that served as regressors were: gender, age, BMI Z-score, days of stay, number of administered drugs, presence of ADRs and diagnoses (C00-D48 Neoplasms, Q00-Q99 Congenital Malformation, D50-D89 Dis. of Blood, Blood-forming Organs and Immunity and G00-G99 Dis. of Nervous System).

Statistical analysis was performed using the Stata Program (version 13). In all cases, a *p*-value < 0.05 was considered to be statistically significant.

### Ethical approval

The study was approved by the HIMFG Research Commission, Ethics and Biosafety Committees with authorization number HIM 2017–008”. It is important mentioning that no information identifying the patients, such as names, initials or institutional registry number will be disclosed in the publication or the database.

## Results

Of 915 patients that were included in this analysis, 61% had at least 1 potential DDI, with a median of 4 (2–8). The percentage of patients with at least one Contraindicated, Serious, Significant and Minor potential DDI was 1% (6/556*100), 28% (154/556*100), 85% (475/556*100) y 70% (387/556*100), respectively. Patients with no potential DDIs and those with at least one were statistically different in terms of gender, number of prescribed drugs, days of stay, presence of ADRs, and diagnoses (Table 1).

**Table 1:** Demographic characteristics of patients included in the study.

	At least 1 potential DDI n = 556	No potential DDIs n = 359	*p* value
**Age**	81.32 (75.98–86.67)	83.15 (76.76–89.53)	0.055
Infants (0–23 months)	137 (24.7%)	74 (20.7%)	0.263
Children (2–11 years)	283 (51.0%)	201 (56.1%)	
Adolescents (12–17 years)	136 (24.3%)	84 (23.2%)	
**Gender**			
Female	283 (50.9%)	145 (40.4%)	0.002*
Male	273 (49.1%)	214 (59.6%)	
**Height (m)**	1.0 (1.0–1.0)	1.1 (1.0–1.1)	0.185
**Weight (kg)**	21.97 (20.61–23.32)	23.86 (22.10–25.63)	0.076
**BMI Z-score**			
Obesity	33 (5.9%)	21 (5.9%)	0.418
Overweight	50 (9.0%)	40 (11.2%)	
Normal	260 (46.8%)	177 (49.6%)	
Malnutrition	212 (38.2%)	119 (33.3%)	
**Number of administered drugs**	7 (5–10)	3 (2–5)	< 0.001*
**Days of stay**	3 (2–5)	2 (1–4)	0.033*
**Presence of ADRs**			
Yes	149 (26.8%)	75 (20.9%)	0.049*
No	407 (73.2%)	284 (79.1%)	
**Diagnoses**			
C00-D48 Neoplasms	138 (25.0%)	84 (23.6%)	< 0.001*
D50-D89 Dis. of Blood, Blood-forming Organs and Immunity	52 (9.4%)	89 (25.0%)	
G00-G99 Dis. of Nervous System	46 (8.3%)	11 (3.0%)	
Q00-Q99 Congenital malformations	114 (20.7%)	43 (12.0%)	
Other^**a**^	206 (37.0%)	132 (36.7%)	

a: A00-B99 Infectious and Parasitic Dis., E00-E90 Endocrine, Nutritional and Metabolic Dis., F00-F99 Mental and Behavioral Disord., H00-H59 Dis. Eye and adnexa, I00-I99 Dis. Circulatory Syst., J00-J99 Dis. Respiratory Syst., K00-K93 Dis. Digestive Syst., L00-L99 Dis. Skin, M00-M99 Dis. Musculoskeletal Syst., N00-N99 Dis. Genitourinary Syst., P00-P96 Perinatal Origin, R00-R99 Not classified, S00-T98 Trauma, Poisoning and external causes, V01-Y98 External causes of morbidity-mortality, Z00-Z99 Health services.

Category of age, Gender, Category of BMI Z-score, Diagnoses, Presence of ADRs: n (%).

Age, height, weight: mean (95% CI).

Number of drugs, Days of stay: median (25-75^th^ percentiles).

* *p* < 0.05.

A total of 3,631potential DDIs were identified in all 915 patients, out of which 0.2% were “Contraindicated”, 7.5% were “Serious-Use Alternative”, 62.8% were “Significant-Monitor Closely” and 29.5% were “Minor”. S1 Table shows the 10 most common drug combinations involved in potential DDIs according to each severity category.

Table 2 shows the results of the two strategies to select the models with the best fit. The α statistic has a value of 0 when there is data equidispersion, and different than 0 otherwise (data overdispersion). The LR test was used to prove the null-hypothesis: H_0_: α = 0, which was rejected for the potential DDIs and significant potential DDIs variables, indicating the presence of overdispersion and pointing at ZINB as the model with the best fit. For the serious potential DDIs and minor potential DDIs variables, the null-hypothesis could not be rejected, which suggests that it is possible for the assumption of equal dispersion required for using the ZIP model to be sustained. The goodness of fit values (AIC and BIC) are shown to confirm the choice between the ZIP and the ZINB models. The model with the lowest AIC and BIC values was chosen. The number of contraindicated potential DDIs variable does not appear in Table 3 since the number of events was extremely low, which hinders estimation of any model.

**Table 2:** Goodness of fit evaluation to select the best model for count data.

Dependent variable	α value	LR test	Statistic	ZIP	ZINB
Total potential DDIs	0.38	LR test: chibar2 = 204.28 (p<0.001)	BIC	3943.813	3526.375
			AIC	3828.689	3406.454
Serious potential DDIs	1.61 X 10^−8^	LR test: chibar2 = 1.8 X 10^−5^ (p = 0.4983)	BIC	482.589	488.357
			AIC	399.686	401.686
Significant potential DDIs	0.37	LR test: chibar2 = 131.57 (p<0.001)	BIC	1819.443	1694.099
			AIC	1726.416	1596.843
Minor potential DDIs	0.039	LR test: chibar2 = 1.15 (p = 0.1414)	BIC	1142.09	1147.059
			AIC	1051.395	1052.242

ZIP: Zero-inflated Poisson model.

ZINB: Zero-inflated negative binomial model.

BIC: Bayesian information criteria.

AIC: Akaike information criteria.

LR test: Likelihood ratio test.

**Table 3:** Zero-inflated count regression models results.

	Total potential DDIs (ZINB Model)		Serious potential DDIs (ZIP Model)		Significant potential DDIs (ZINB Model)		Minor potential DDIs (ZIP Model)	
**Covariate (Count Equation-Not always 0)**	**exp (β)** n = 556	***p* value**	**exp (β)** n = 154	***p* value**	**exp (β)** n = 475	***p* value**	**exp (β)** n = 387	***p* value**
**Gender**								
Male	Ref	-	-	-	-	-	-	-
Female	1.18	0.013*	1.23	0.146	1.21	0.010*	1.01	0.897
**Age (months)**	1.001	0.018*	1.00	0.810	1.001	0.047*	1.001	0.038*
**BMI Z-score**	0.99	0.586	1.04	0.279	0.99	0.829	0.99	0.994
**Days of stay**	0.96	0.004*	0.98	0.265	0.96	0.005*	0.98	0.022*
**Number of administered drugs**	1.22	0.000*	1.08	0.000*	1.20	0.000*	1.12	0.000*
**Presence of ADRs**								
No	Ref	-	-	-	-	-	-	-
Yes	0.92	0.457	1.14	0.491	0.87	0.224	1.19	0.065
**Diagnoses**								
Other^a^	Ref	-	-	-	-	-	-	-
C00-D48 Neoplasms	0.67	0.001*	0.65	0.088	0.59	0.000*	0.75	0.007*
Q00-Q99 Congenital malformations	1.24	0.019*	1.36	0.089	1.32	0.005*	1.10	0.333
D50-D89 Dis. of Blood, Blood-forming Organs and Immunity	0.76	0.041*	0.71	0.308	0.78	0.094	0.79	0.136
G00-G99 Dis. of Nervous System	1.31	0.029*	0.88	0.641	1.08	0.537	1.57	0.000*
**Covariate (Binary Equation- Always 0)**	**exp (β)** n = 359	***p* value**	**exp (β)** n = 359	***p* value**	**exp (β)** n = 359	***p* value**	**exp (β)** n = 359	***p* value**
**Gender**								
Male	Ref	-	-	-	-	-	-	-
Female	0.86	0.610	1.15	0.837	1.26	0.553	0.49	0.056
**Age (months)**	0.99	0.710	0.98	0.003*	0.99	0.814	0.99	0.412
**BMI Z-score**	0.99	0.958	0.88	0.458	1.04	0.552	1.04	0.676
**Days of stay**	1.09	0.171	0.95	0.713	1.14	0.167	1.08	0.325
**Number of administered drugs**	0.35	0.000*	0.21	0.000*	0.19	0.000*	0.28	0.000*
**Presence of ADRs**								
No	Ref	-	-	-	-	-	-	-
Yes	1.78	0.335	2.06	0.498	5.69	0.040*	1.60	0.504
**Diagnoses**								
Other^a^	Ref	-	-	-	-	-	-	-
C00-D48 Neoplasms	1.26	0.694	17.07	0.027*	1.46	0.641	0.95	0.942
Q00-Q99 Congenital malformations	0.31	0.003*	0.31	0.192	0.21	0.003*	0.35	0.049*
D50-D89 Dis. of Blood, Blood-forming Organs and Immunity	1.08	0.834	3.42	0.270	0.77	0.631	1.31	0.619
G00-G99 Dis. of Nervous System	0.16	0.035*	0.02	0.031*	0.01	0.196	0.28	0.141

a: A00-B99 Infectious and Parasitic Dis., E00-E90 Endocrine, Nutritional and Metabolic Dis., F00-F99 Mental and Behavior Disord., H00-H59 Dis. Eye and Adnexa, I00-I99 Dis. Circulatory Syst., J00-J99 Dis. Respiratory Syst., K00-K93 Dis. Digestive Syst., L00-L99 Dis. Skin, M00-M99 Dis. Musculoskeletal Syst., N00-N99 Dis. Genitourinary Syst., P00-P96 Perinatal Origin, R00-R99 Not classified, S00-T98 Trauma, Poisoning and external cause, V01-Y98 External causes of morbidity-mortality, Z00-Z99 Health services.

* *p* < 0.05.

Table 3 shows the results of the regressions for the four models adjusted for the potential DDIs variable (total and stratified by severity), with coefficients being exponentially expressed in order to be interpreted as the change in the response variable (expressed in odds) per increase unit for each one of the covariates and for both groups of the model (Count equation-not always 0 and Binary equation-always 0). The first regression model shown in Table 3 is a ZINB model that used potential DDIs total number as response variable. In the Count equation-not always 0 group, the covariates with a positive and statistically significant association were the female gender, age, the number of administered drugs, congenital malformations (Q00-Q99) and diseases of the nervous system (G00-G99), whereas those with a negative and statistically significant association were ED days of stay, neoplasms (C00-D48) and diseases of blood (D50-D89). On the other hand, in the Binary Equation-Always 0 group, the number of administered drugs, congenital malformations (Q00-Q99) and diseases of the nervous system (G00-G99) had a negative and statistically significant association. The second regression model (the ZIP model), accounted for the number of serious potential DDIs based on the same covariate vector than the first model. In the Count equation-not always 0 group, the number of administered drugs had a positive and statistically significant association, whereas in the Binary equation-Always 0 group, the age, the number of administered drugs and diseases of the central nervous system (G00-G99) had a negative and statistically significant association, in contrast with neoplasms (C00-D48), which showed a positive and statistically significant association. In the third regression model (the ZINB model), the number of significant potential DDIs was accounted for. Similar results to those observed in the first model were found (potential DDIs total number), both in magnitude and direction of associations in both groups of the model (Count Equation-Not always 0 and Binary Equation-Always 0). It is important highlighting that only in the Binary Equation-Always 0 group of this group did the presence of ADRs exhibit a positive and statistically significant association. The results of the regression model (ZIP model) used to predict the number of minor potential DDIs are similar to those in the total number of potential DDIs model, with the difference that in the Binary Equation-Always 0 group only the number of administered drugs and congenital malformations (Q00-Q99) had a negative and statistically significant association.

## Discussion

The 61% prevalence of potential DDIs found in this study is within the range of values reported in children (from 3.8% to 75%) [10–16]. As it can be appreciated, there is wide variability in the potential DDIs prevalence values reported in the literature, which can be explained by **a)** the included population, **b)** the study design and **c)** the software used for their identification. It is important to highlight that prevalence in this work (61%) is very similar to that reported in a study that only included onco-hematological pediatric patients (56.7%) [14] in spite of the study design and the used software being different, which can be explained by the high resemblance of the study populations in both works, since onco-hematological conditions are among the main causes the HIMFG provides care for [27]. Particularly, children admitted to EDs have critical medical conditions that make them more susceptible to the administration of multiple drugs, to complex treatment regimens, to long ED stays and to care by different specialist physicians for consultations [19–20]. There are more than 54 different softwares for DDIs assessment [28], but according to a systematic review that included articles of children and adults between 1976 and 2014, the most widely used are the Drug-Relax software from Micromedex® Healthcare Series, Drug Interactions Facts®, Lexi-Interact®, Pharmavista®, EpocratesRx®, MediQ® and Drug interaction checker® [29]. The reason why in this study the Medscape Drug Interaction Checker software was selected is because we consider it to be highly likely to be used in clinical practice owing to its simple and free access in addition to being easy to use and having good structural quality [28]. The present study is important because, to our knowledge, it is the first one carried out in a pediatric ED using the Medscape Drug Interaction Checker software for potential DDIs assessment, since only one article in children with HIV was identified using it as well [13]. As previously mentioned, the potential DDIs concept refers to the “*possibility*” a drug has to alter the effects of another when both are simultaneously administered, i.e., the clinical consequence does not always occur to patients. In the literature review, a predominance of studies reporting only potential DDIs was identified, which makes for DDIs that occur and harm inflicted on patients difficult to be estimated. In adults, DDIs have been reported to cause 1.1% of hospital admissions and 0.1% of hospital visits [5], whereas in cancer patients they cause 2% of admissions [30]. In pediatric population, two publications were identified reporting DDIs impact on patients, with the first one mentioning that they cause 0.78% of admissions [31], whereas the second refers they cause 0% of ED consultations [32]. There are only few studies assessing potential DDIs in hospitalized adult patients that have reviewed patient charts to identify DDIs that actually occur (real DDIs). In this case, three publications were identified reporting potential DDIs frequencies of 7.7%, 4.7% and 11.1%, while reported DDI frequencies were 0.7%, 0.1% and 1%, respectively [33–35]. In another study of hospitalized patients, there was 0.95% of DDIs, without the percentage of potential DDIs being mentioned [36]. To our knowledge, there are no studies conducted in children assessing potential DDIs and DDIs frequency, and the relevance of potential DDIs in the clinical context may therefore come to be controversial, which in addition leaves an important line of investigation open. The potential DDIs severity classification (Contraindicated, Serious, Significant and Minor) can help doctors to identify potential DDIs clinical manifestations that require additional care and surveillance, since treatment efficacy can be compromised if the use of certain drugs is avoided owing to unfounded potential DDIs. For example, it can be stated that, in the present study, 29.5% of clinical manifestations are highly unlikely to occur or to be clinically relevant since they were classified as “Minor”. Therefore, the decision on actions to be taken by doctors to minimize potential DDIs impact on their patients should be determined on an individual basis, and this requires careful evaluation of the risk-benefit ratio between treatment discontinuation vs. continuation but with close monitoring. It is difficult for comparisons of potential DDIs severity classification to be established with other studies since, as previously mentioned, there are numerous softwares for their assessment and there is no standardization of the terminology used to classify severity [37]; however, it is important mentioning that some potential DDIs that were identified are consistent with those in other studies that used different softwares for their assessment, for example: linezolid+norepinephrine [12,16], ceftriaxone+calcium gluconate [12] and furosemide+amikacin [14]. An elevated discrepancy has been reported between the number of DDIs detected with electronic systems and the number of clinically relevant DDIs evaluated by doctors, which might favor the likelihood for prescribing physicians to ignore information on the possibility of DDIs occurrence [29]. However, in clinical practice, it will always be important being informed about potential DDIs, out of which Table 3 offers the most relevant examples found in pediatric EDs.

To the best of our knowledge, this is the first study to estimate the magnitude of the covariates effect on the number of observed events (total and severity-stratified potential DDIs) using a statistical model that is appropriate for count variables such as ZIP and ZINB. In the Count Equation-Not always 0 group of first model (Total potential DDIs), the fact that girls have 1.18 higher odds of having a potential DDI than boys is opposite to previous reports [14], and it is therefore important to carry out further studies on this subject in pediatrics. On the other hand, the association of age with potential DDIs has already been previously reported [10]. One finding that drew our attention, because it is opposite to previous observations reported in PICU patients [16], is the effect days of hospital stay have on potential DDIs, since for each additional day of ED stay, the probability of a potential DDI is reduced by 4%, which might be explained by the fact that, during the first days of hospital stay, the likelihood for a potential DDI to occur is higher (S2 Fig), since owing to the serious condition of patients taken care of at the ED, physicians have to prioritize decision making to address the emergency and save the patient’s life, leaving pharmacological aspects related to potential DDIs for a later moment. The observed effect by the number of administered drugs was as expected, according to observations reported in other studies [14,16]. This can be observed in the Count Equation-Not always 0 model where, for each additional administered drug, the odds of a larger number of potential DDIs to occur are increased by 1.22, whereas in the Binary Equation-Always 0 group, for each additional administered drug, the odds of belonging to the group with no potential DDIs (Binary Equation-Always 0) are reduced by 65%. Another finding that stands out is the group of Tumors (C00-D48) coefficient, since in contrast with previous reports [10,14,16], in the present study, patients admitted to the ED with this diagnosis have a 33% decrease in the odds of having potential DDIs, in comparison with patients belonging to the “Other diagnoses” group, which might be explained by the fact that the HIMFG provides care mainly to cancer patients, in whom the main cause for ED admission is febrile neutropenia, the treatment of which is highly standardized between physicians. A similar situation is observed in the group of diseases of the blood (D50-D89), since the main reason for admission to the ED are hemorrhagic episodes in patients with hemophilia, the treatment protocol of which limits multi-drug therapy and is perfectly defined. In patients with Congenital Malformations and Diseases of the Nervous System, an increase was observed in the odds of potential DDIs in comparison with the Other Diagnoses group, which has already been previously reported [10,16]. On the other hand, in the Binary Equation-Always 0 of first group (total potential DDIs), the covariates that were statistically significant and had exponential coefficients lower than 1 were the number of administered drugs, congenital malformations (Q00-Q99) and nervous system diseases (G00-G99), which is consistent with the direction of the association observed in the Count Equation-Not always 0 group, where they showed increased odds (> 1). As previously mentioned in the Results section, in the Count equation-Not always 0 group of the second model (serious potential DDIs), only the number of administered drugs was statistically significant, which may be explained by the low percentage of patients (28%) who had at least one serious potential DDI. In the Binary equation-Always 0 group, the negative association of age (for each month of age increase, the odds of belonging to the “no potential DDIs group” are reduced by 2%) stands out, as it might suggest that ED physicians administer more drugs to older than to small children. Similarly, the fact that patients in the Neoplasms group are highly likely to belong to the group with no serious potential DDIs (exp(β) = 17.07) stands out, since, as previously mentioned, the treatment of febrile neutropenia at the ED is highly standardized between doctors, in contrast with the treatment of patients with diseases of the nervous system (exp(β) = 0.02). As previously mentioned, the results of the regression model for the significant potential DDIs variable were very similar to those observed in the first model (potential DDIs total number), which might be explained by the fact that in 85% of patients who had at least one potential DDIs it was significant. In the Binary Equation-Always 0 group, the effect of the presence of ADRs (exp(β) = 5.69) stands out, since it was not evident in the other models and it might suggest that patients with ADRs are more likely not to have significant potential DDIs in comparison with those with no ADRs. This is important, since patients exposed to potential DDIs have been reported to have ADRs [38], which highlights the importance of conducting prospective studies evaluating potential DDIs clinical importance in children, using at least patient medical records. Likewise, the results of the regression model for the minor potential DDIs variable were very similar to those observed in the first model (total potential DDIs), which could be explained by the fact that in 70% of patients who had at least one potential DDI it was minor. The different models that were used in this work to account for potential DDIs stratified by severity, allowed for some effects that are different between serious and significant potential DDIs to be identified.

### Study limitations

The limitations of the study that should be taken into consideration when interpreting the results include:

- The study was not focused on potential DDIs clinical consequences, but on their potential occurrence
- The results cannot be extrapolated to other hospital areas
- Since data were taken only at one point in time, it is not possible for causality inferences to be made.
- It is possible that if the same population is examined at other moment, the obtained results may be different because the information was collected only once
- There may be a selection bias because the intervention study included patients with complete information on their medical records

## Conclusion

The study demonstrates that the prevalence of potential DDIs at the ED is high and that establishing strategies to monitor the safety of patients during their stay at the ED is therefore necessary, even though physicians have taken actions in younger children, in those with ADRs, and in those diagnosed with neoplasms and diseases of the blood. In addition, the importance of conducting investigations to estimate the real harm potential DDIs cause in patients is suggested.

## Supporting information

S1 Fig Frequency distribution of the “potential DDIs” variable. (TIF)

S2 Fig Correlation between ED length of stay (days) and potential DDIs. (TIF)

S1 Table Top 10 most common potential DDIs stratified according to severity. (DOCX)

S2 Table Original data used for the data analysis. (XLSX)

S1 File Original protocol which was reviewed by the Institutional Review Board. (PDF)

S2 File Letter of approval by the Institutional Review Board. (PDF)
