# Supplementary figures and images for: Potential drug-drug interactions and their risk factors in pediatric patients admitted to the emergency department of a tertiary care hospital in Mexico
Source: PLoS One. 2018 Jan 5;13(1):e0190882. doi: 10.1371/journal.pone.0190882 (PMC5755936; doi:10.1371/journal.pone.0190882)

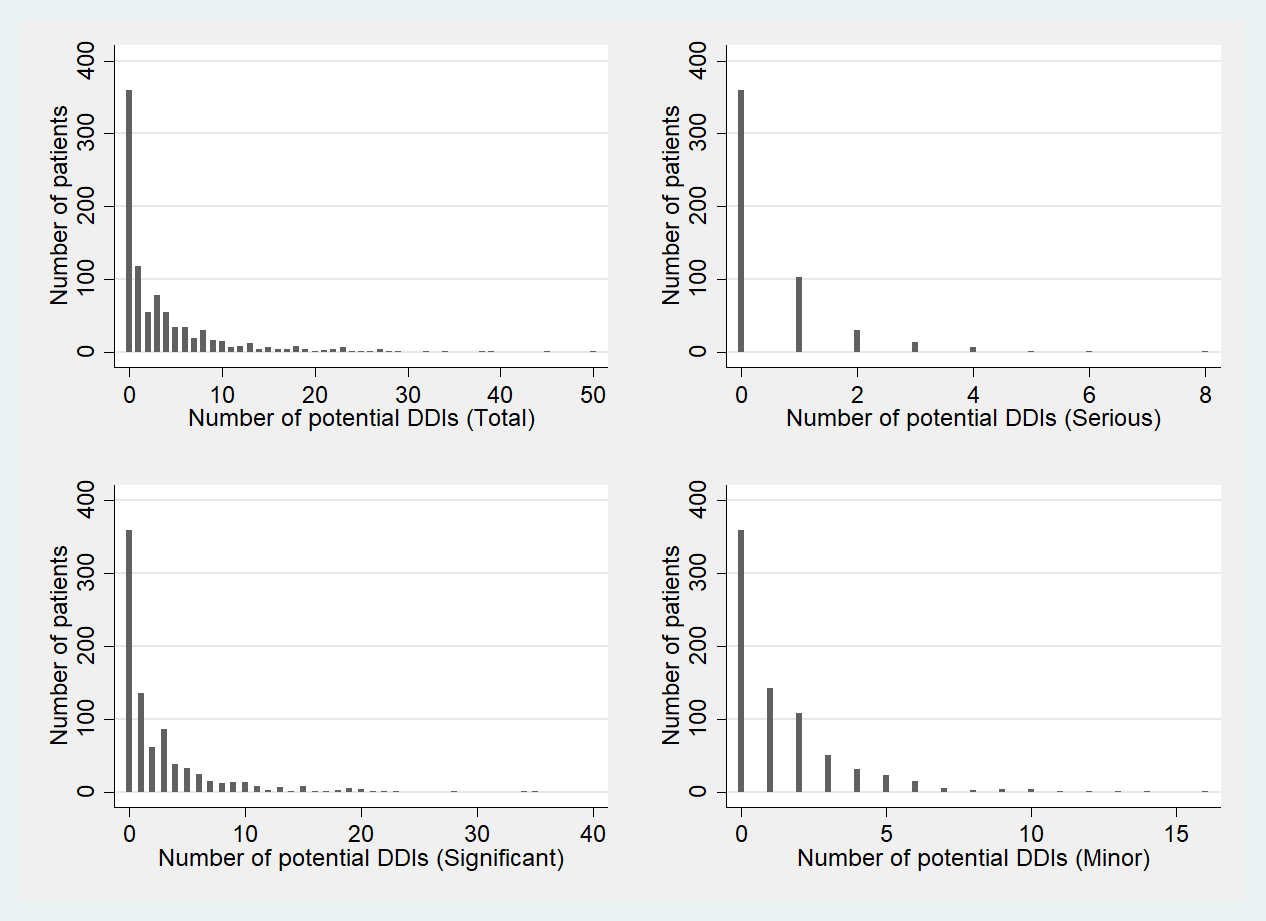

Supplement: S1 Fig — (TIF) [file pone.0190882.s001.tif]

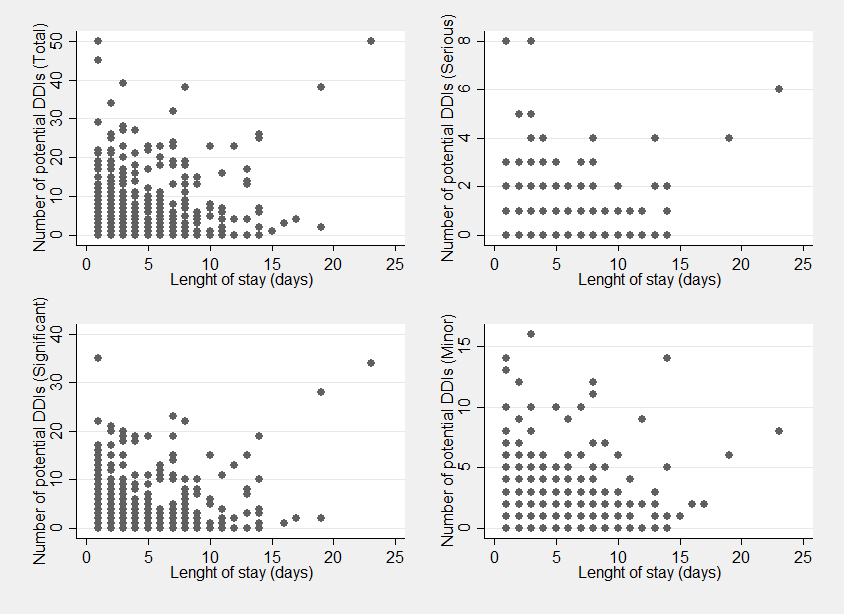

Supplement: S2 Fig — (TIF) [file pone.0190882.s002.tif]
