# Supplementary material for: Potential drug-drug interactions and their risk factors in pediatric patients admitted to the emergency department of a tertiary care hospital in Mexico
Source: PLoS One. 2018 Jan 5;13(1):e0190882. doi: 10.1371/journal.pone.0190882 (PMC5755936; doi:10.1371/journal.pone.0190882)
Supplement: S1 Table — (DOCX) [file pone.0190882.s003.docx]

| **S1 Table.** Top 10 most common potential DDIs stratified according to severity | | | |
| --- | --- | --- | --- |
| **Contraindicated** | **n/N=**  **7/3,631**  **(0.2%)** | **Mechanism** | **Summary of potential adverse consequences** |
| Amikacin+Amphotericin B Deoxycholate | 3 (42.9%) | NR | Both increase nephrotoxicity and/or ototoxicity |
| Linezolid+Adrenergic Agonists (Dobutamine/ Norepinephrine) | 2 (28.6%) | PD | Risk of acute hypertensive episode |
| Ceftriaxone+Calcium Gluconate | 1 (14.3%) | NR | Do not use ANY calcium-containing solutions (including Ringer's or Harmann's) in combination with IV ceftriaxone; risk of potentially fatal particulate precipitation in lungs, kidneys. Separate by at least 48 hrs |
| Cisapride+Octreotide | 1 (14.3%) | NR | Both increase QTc interval |
| **Serious-Use alternative** | **n/N=**  **272/3,631**  **(7.5%)** | **Mechanism** | **Summary of potential adverse consequences** |
| Furosemide+Amikacin | 29 (10.7%) | PD | Possible serious or life-threatening interaction. Monitor closely. Increased risk of ototoxicity and nephrotoxicity |
| Fentanyl+Ketamine | 20 (7.4%) | PD | Possible serious or life-threatening interaction. Monitor closely. Co-administration with other CNS depressants, such as skeletal muscle relaxants, may cause respiratory depression, hypotension, profound sedation, coma, and/or death. Consider dose reduction of either or both agents to avoid serious adverse effects. Monitor for hypotension, respiratory depression, and profound sedation |
| Fentanyl+Vecuronium | 17 (6.3%) | PD | Possible serious or life-threatening interaction. Monitor closely. Co-administration with other CNS depressants, such as skeletal muscle relaxants, may cause respiratory depression, hypotension, profound sedation, coma, and/or death. Consider dose reduction of either or both agents to avoid serious adverse effects. Monitor for hypotension, respiratory depression, and profound sedation |
| Ketamine+Norepinephrine | 13 (4.8%) | PK | High likelihood of serious or life-threatening interaction. Contraindicated unless benefits outweigh risks and no alternatives are available. Never use combination |
| Mycophenolate+Tacrolimus | 10 (3.7%) | NR | Both mycophenolate and tacrolimus increase immunosuppressive effects; risk of infection. High likelihood of serious or life-threatening interaction. Contraindicated unless benefits outweigh risks and no alternatives are available |
| Losartan+Captopril | 9 (3.3%) | PD | Possible serious or life-threatening interaction. Monitor closely. Dual blockade of renin-angiotensin system increases risks of hypotension, hyperkalemia, and renal impairment |
| Amikacin+Vecuronium | 7 (2.6%) | PD | Possible serious or life-threatening interaction. Monitor closely. Risk of apnea |
| Azathioprine+Hydroxy­chloroquine sulfate | 7 (2.6%) | NR | Both azathioprine and hydroxy­chloroquine sulfate increase immunosuppressive effects; risk of infection. High likely hood of serious or life-threatening interaction. Contraindicated unless benefits outweigh risks and no alternatives are available |
| Carbamazepine+Diazepam | 7 (2.6%) | PK | Possible serious or life-threatening interaction. Monitor closely |
| Ketamine+Epinephrine | 6 (2.2%) | NR | Possible serious or life-threatening interaction. Monitor closely |
| **Significant-Monitor Closely** | **n/N= 2,281/3,631**  **(62.8%)** | **Mechanism** | **Summary of potential adverse consequences** |
| Spironolactone+Furosemide | 104 (4.5%) | NR | Spironolactone increases and furosemide decreases serum potassium. Effect of interaction is not clear, use with caution. |
| Captopril+Furosemide | 68 (3.0%) | PD | Risk of acute hypotension, renal insufficiency |
| Captopril+Spironolactone | 64 (2.8%) | PD | Risk of hyperkalemia |
| Ketamine+Midazolam | 61 (2.7%) | NR | Both increase sedation |
| Dobutamine+Norepinephrine | 42 (1.8%) | NR | Both dobutamine and norepinephrine decrease serum potassium |
| Trimethoprim+Potassium Chloride | 41 (1.8%) | PK | Both trimethoprim and potassium chloride increase serum potassium. May cause hyperkalemia, particularly with high doses, renal insufficiency, or when combined with other drugs that cause hyperkalemia |
| Spironolactone+Digoxin | 31(1.4%) | PK | Spironolactone has been shown to increase digoxin half-life and subsequent toxicity may occur. |
| Amikacin+Vancomycin | 26 (1.1%) | NR | Both amikacin and vancomycin increase nephrotoxicity and/or ototoxicity |
| Midazolam+Amikacin | 25 (1.1%) | PK | Midazolam will decrease the level or effect of amikacin |
| Midazolam+Dobutamine | 25 (1.1%) | NR | Midazolam increases and dobutamine decreases sedation. Effect of interaction is not clear, use caution |
| **Minor** | **n/N=**  **1,071/3,631 (29.5%)** | **Mechanism** | **Summary of potential adverse consequences** |
| Omeprazole+Sulfamet­hoxa­zole | 42 (3.9%) | PK | Omeprazole will increase the level or effect of sulfamethoxazole |
| Ibuprofen+Amikacin | 38 (3.5%) | PK | Ibuprofen increases levels of amikacin by decreasing renal clearance. Interaction mainly occurs in preterm infants |
| Omeprazole+Midazolam | 37 (3.5%) | PK | Omeprazole increases levels of midazolam by decreasing metabolism |
| Cefuroxime+Furosemide | 28 (2.6%) | PD | Cefuroxime increases toxicity of furosemide by synergism. Increased risk of nephrotoxicity |
| Metronidazole+Acetami­nophen | 24 (2.2%) | PK | Metronidazole will increase the level or effect of acetaminophen |
| Dexamethasone+Omeprazole | 23 (2.1%) | PK | Dexamethasone will decrease the level or effect of omeprazole |
| Valproic Acid+Acetaminophen | 23 (2.1%) | PK | Valproic acid decreases levels of acetaminophen by increasing metabolism. Enhanced metabolism increases levels of hepatotoxic metabolites |
| Cefepime+Furosemide | 22 (2.1%) | PD | Cefepime increases toxicity of furosemide by synergism. Increased risk of nephrotoxicity |
| Phenytoin+Acetaminophen | 22 (2.1%) | PK | Phenytoin decreases levels of acetaminophen by increasing metabolism. Enhanced metabolism increases levels of hepatotoxic metabolites |
| Sulfamethoxazole+Ibuprofen | 21 (2.0%) | PK | Sulfamethoxazole will increase the level or effect of ibuprofen |
| NR: Mechanism not reported in Medscape Drug Interaction Checker  PK: Pharmacokinetic Mechanism  PD: Pharmacodynamic Mechanism | | | |
