# Supplementary material for: Potential drug-drug interactions and their risk factors in pediatric patients admitted to the emergency department of a tertiary care hospital in Mexico
Source: PLoS One. 2018 Jan 5;13(1):e0190882. doi: 10.1371/journal.pone.0190882 (PMC5755936; doi:10.1371/journal.pone.0190882)
Supplement: S1 File — (PDF) [file pone.0190882.s005.pdf]

HOSPITAL INFANTIL DE MÉXICO FEDERICO GÓMEZ

Departamento de Evaluación y Análisis de Medicamentos

Título del trabajo:

Estudio fármaco epidemiológico de potenciales interacciones fármaco-fármaco en niños hospitalizados en un Departamento de Urgencias de un Hospital Pediátrico de tercer nivel en México

Investigador Responsable
Dra. Olga Magdala Morales Ríos

Investigador Suplente
Dr. Luis Jasso Gutiérrez

Investigadores Participantes
Dr. Alfonso Reyes López
Dr. Juan Garduño Espinosa
Dr. Onofre Muñoz Hernández

Ciudad de México, Agosto del 2016

I. ÍNDICE

I. ÍNDICE 2
II. RESUMEN 3
III. ANTECEDENTES 5
V. PLANTEAMIENTO DEL PROBLEMA Y JUSTIFICACIÓN 6
VI. PREGUNTAS DE INVESTIGACIÓN 7
VII. HIPÓTESIS 7
VIII. OBJETIVOS 7
IX. METODOLOGÍA Y PLAN DE ANÁLISIS DE LOS DATOS 8
X. LIMITACIONES DEL ESTUDIO 15
XI. BIBLIOGRAFÍA 16
XII. ANEXO 1 18

II. RESUMEN

Antecedentes:A pesar de que en la práctica clínica diaria no todas las Interacciones Fármaco-Fármaco (IF-F) detectadas en un paciente pueden llegar a desarrollarse (potenciales IF-F), es idealmente necesario,la identificación de las potenciales IF-F debido a que su impacto puede incrementar el riesgo de Reacciones Adversas a Medicamentos (RAMs), su toxicidad o pérdida de eficacia del tratamiento. En la población pediátrica la prevalencia de potenciales IF-F es elevada en pacientes hospitalizados (49%), con VIH (67.1%), en unidades de cuidados intensivos (75%) y con algún diagnóstico hemato-oncológico (56.7%). En niños se ha encontrado asociación con la edad, el número de prescripciones por visita, el número de visitas por año, algunos diagnósticos, el número de medicamentos, los días de estancia y ser de raza blanca. Los niños hospitalizados en Urgencias son vulnerables a presentar potenciales IF-F ya que pueden recibir hasta más de 25 medicamentos durante su estancia. En México, sólo hay dos publicaciones realizadas en pacientes adultos, en las que se estudiaron las potenciales IF-F y sus factores asociados, y en el caso de los niños no se encontraron publicaciones al respecto. Por otra parte, internacionalmente las publicaciones en los niños son escasas y sólo en tres de ellas se han estudiado factores asociados.

Objetivos

a).- Estimar la prevalencia de las potenciales IF-F en pacientes pediátricos hospitalizados en un departamento de urgencias de un hospital pediátrico de tercer nivel en México

b).- Describir las características de las potenciales IF-F en pacientes pediátricos hospitalizados en un departamento de urgencias de un hospital pediátrico de tercer nivel en México

c).- Investigar si la Edad, Sexo, Estatura, Peso, Puntaje Z de IMC, Número de medicamentos, Días de estancia, Condición al egreso, Presencia de RAM y algunos Diagnósticos, se asocien a la presencia de potenciales IF-F en pacientes pediátricos hospitalizados en un departamento de urgencias de un hospital pediátrico de tercer nivel en México

Metodología y plan de análisis de los datos

Diseño: Se realizó un análisis secundario de datos de 1,179 pacientes que ingresaron al Departamento de Urgencias del HIMFG.

Muestreo: No probabilístico por conveniencia

Variable dependiente:Número de potenciales IF-F

Covariables: Edad, sexo, talla, peso, Z de IMC, número de medicamentos, diagnósticos de base, presencia de RAMs y condición al egreso

Procedimientos: Previamente se revisó la calidad de la información de la base de datos y para la evaluación e identificación de las potenciales interacciones se utilizará el programa de Mescape Online. El análisis se realizará ingresando los medicamentos prescritos en cada uno de los pacientes.

Plan de análisis estadístico:

- Análisis descriptivo: número de casos (%), media (IC 95%) y mediana (percentiles 25-75)
- Análisis bivariado: Se contrastarán cada una de las covariables entre los pacientes que presentaron al menos una potencial IF-F y los que no.
- Análisis multivariado: Regresiones para Datos de Conteo

Se considerará $p < 0.05$ como estadísticamente significativa y se utilizaráSPSS versión 18 y STATA versión 13

III. ANTECEDENTES

Los medicamentos son una de las principales herramientas terapéuticas y su empleo resulta esencial en la mayor parte de los actos médicos. No obstante, la prescripción de varios medicamentos puede favorecer la presencia de interacciones fármaco-fármaco (IF-F), las cuales pueden identificarse cuando la respuesta farmacológica o clínica a la administración de una combinación de medicamentos es diferente de la que se espera por los efectos conocidos de los dos medicamentos prescritos de manera individual[1]. A pesar que en la práctica clínica diaria no todas las IF-F detectadas en un paciente pueden llegar a desarrollarse (potenciales IF-F) su identificación es relevante debido a que su impacto puede ser el incremento del riesgo de Reacciones Adversas a Medicamentos (RAMs), toxicidad o pérdida de eficacia del tratamiento[2].

En lospacientes adultosno sólo se han investigado las potenciales IF-F si no que ya existen varias publicaciones que reportan los eventos adversos que causaron las IF-F, por ejemplo en una revisión sistemática que incluyó 14 artículos desde el año de 1994 hasta el 2010 cuyo objetivo fue estimar la prevalencia de admisiones o visitas al hospital asociadas con una IF-F, se encontró que la prevalencia para ingresos hospitalarios debido a una potencial IF-F fue del 1.1% y en el caso de las visitas al hospital por dicha causa fue de 0.1%[3].Otro estudio realizado en el Reino Unido estimó que el 16.6% de las RAMs que fueron causa de ingresos hospitalarios estuvieron asociados con IF-F[4].

En el caso de los pacientes pediátricos el enfoque de las IF-F requiere consideraciones diferentes a los adultos ya que: **a)** pueden reaccionar de manera diferente al mismo fármaco o a una combinación de los mismos, lo que podría ser explicado por las diferencias en la fisiología de los niños a diferentes edades lo que afectaría la farmacocinética[5], **b)** la prescripción de medicamentos unlicensed (medicamentos que no están autorizados por las Agencias Reguladoras para su uso en humanos, por ejemplo medicamentos preparados por la farmacia del hospital o por cualquier farmacia), y los off label (medicamentos cuyos usos no están detallados en el resumen de características del producto como indicación terapéutica, dosis, y forma farmacéutica)[6] y **c)** los niños hospitalizados pueden estar expuestos a más de 25 medicamentos con estancias prolongadas[7].

En la población pediátrica la prevalencia de potenciales IF-F es elevada en pacientes hospitalizados (49%)[8]; en los enfermos con VIH (67.1%)[9]; en unidades de cuidados intensivos (75%)[10]; o con algún diagnóstico hemato-oncológico (56.7%)[11]. Las variaciones entre las

prevalencias se pueden explicar por las diferencias en el diseño, la población de pacientes y por el software utilizado para identificarlas. Se encontraron algunas publicaciones que estudian los factores asociados a las potenciales IF-F en niños. En los hospitalizados se encontró asociación con la edad de los pacientes, el número de prescripciones por visita, el número de visitas por año y algunos diagnósticos (epilepsia, leucemia o artritis reumatoide)[11-12]. Otro estudio realizado en niños de unidades de cuidados intensivos identificó asociación con algunos diagnósticos (neoplasias, sistema circulatorio, anomalías congénitas y enfermedades del sistema nervioso), con la presencia de condiciones complejas crónicas, con el número de medicamentos, con los días de estancia y con la raza blanca[10].

En los años 2012 a 2015 nuestro grupo de investigación realizó el protocolo titulado "Estudio comparativo de las reacciones adversas a medicamentos entre la Farmacovigilancia Intensiva y la Notificación Espontánea en un Departamento de Urgencias de un hospital pediátrico"[13], cuyo objetivo fue evaluar el efecto de una intervención para mejorar el subreporte de Reacciones Adversas a Medicamentos (RAMs) en el Departamento de Urgencias del Hospital Infantil de México Federico Gómez (DptoUrg). La información se obtuvo de la revisión de 1,179 pacientes, en los que se incluyeron las características demográficas, los diagnósticos de base, la presencia de RAMs y los medicamentos prescritos durante su estancia. Con esa información obtenida en el trabajo previo, se planeó en un análisis secundario de los datos, realizar el presente trabajo con el objetivo de evaluar las potenciales IF-F que presentaron los pacientes y algunos factores asociados.

V. PLANTEAMIENTO DEL PROBLEMA Y JUSTIFICACIÓN

Como se mencionó previamente las IF-F incrementan el riesgo de RAMs, toxicidad, pérdida de eficacia en el tratamiento e incremento en los costos;que en el caso de los niños el tema se vuelve más relevante debido a que tienen aspectos fisiológicos y bioquímicos por edades, diferentes a lo que sucede con los adultos, que los hacen potencialmente más susceptibles. Particularmente, los Departamentos de Urgencias son uno de los sitios en los que por la gravedad con la que ingresan los niños, pueden estar sometidos a la administración de hasta más de 25 medicamentos durante su estancia y por lo tanto a que potencialmente puedan tener una frecuencia mayor de potenciales IF-F.En México, hay dos trabajos que estudiaron las potenciales IF-F y sus factores asociadosen adultos[1,14], sin embargo, la información de las frecuencias y características de las potenciales IF-F en los Departamentos de Urgencias de

hospitales pediátricos es muy escasa a nivel internacional y en México es prácticamente inexistente.

VI. PREGUNTAS DE INVESTIGACIÓN

a).- ¿Cuál es la prevalencia de las potenciales IF-F en pacientes pediátricos hospitalizados en un departamento de urgencias de un hospital pediátrico de tercer nivel en México?

b).- ¿Cuáles son las características de las potenciales IF-F en pacientes pediátricos hospitalizados en un departamento de urgencias de un hospital pediátrico de tercer nivel en México?

c).- ¿La Edad, Sexo, Estatura, Peso, Puntaje Z de IMC, Número de medicamentos, Días de estancia, Condición al egreso, Presencia de RAM y algunos Diagnósticos se asocian a la presencia de potenciales IF-F en pacientes hospitalizados en un departamento de urgencias de un hospital pediátrico de tercer nivel en México?

VII. HIPÓTESIS

a).- La prevalencia de las potenciales IF-F en el Departamento de Urgencias de un hospital pediátrico de tercer nivel en Méxicoes mayor al 40%

b).- La Edad, Sexo, Estatura, Peso, Puntaje Z de IMC, Número de medicamentos, Días de estancia, Condición al egreso, Presencia de RAM y algunos Diagnósticos se asocian a la presencia de potenciales IF-F en pacientes hospitalizados en un departamento de urgencias de un hospital pediátrico de tercer nivel en México

VIII. OBJETIVOS

a).- Estimar la prevalencia de las potenciales IF-F en pacientes pediátricos hospitalizados en un departamento de urgencias de un hospital pediátrico de tercer nivel en México

b).- Describir las características de las potenciales IF-F en pacientes pediátricos hospitalizados en un departamento de urgencias de un hospital pediátrico de tercer nivel en México

c).- Analizar si la Edad, Sexo, Estatura, Peso, Puntaje Z de IMC, Número de medicamentos, Días de estancia, Condición al egreso, Presencia de RAM y algunos Diagnósticos se asocian a la presencia de potenciales IF-F en pacientes hospitalizados en un departamento de urgencias de un hospital pediátrico de tercer nivel en México

IX. METODOLOGÍA Y PLAN DE ANÁLISIS DE LOS DATOS

Diseño

Análisis secundario de datos del estudio titulado "Estudio comparativo de las reacciones adversas a medicamentos entre la Farmacovigilancia Intensiva y la Notificación Espontánea en un Departamento de Urgencias de un hospital pediátrico" con número **HIM/2011/037** que fue aprobado por las Comisiones de Investigación, Ética y Bioseguridad del Hospital Infantil de México Federico Gómez el día 3 de mayo de 2012. El diseño del estudio **HIM/2011/037** fue cuasi experimental de antes y después[15] y los objetivos fueron: **a).**Evaluar la efectividad de una intervención integral dirigida a médicos del Departamento de Urgencias y coordinada por un farmacéutico en un hospital pediátrico de tercer nivel de atención sobre la identificación y el reporte de las RAMs y **b).**Evaluar si algunas características de los niños pudieran influir sobre la identificación y reporte de las RAMs que realizan los médicos de un Departamento de Urgencias[13]

Criterios de selección de la población de estudio

Criterios de Inclusión:

- Pacientes que ingresaron al Departamento de Urgencias del HIMFG
- Pacientes a los que durante su estancia se les administraron 2 o más medicamentos

Criterios de exclusión:

- Ninguno

Muestreo y tamaño de muestra

Tamaño de muestra: En el estudio previo**HIM/2011/037**[13]se recolectaron 1,179 pacientes de los cuales 915 cumplen con los criterios de inclusión para este análisis.

Para estimar la prevalencia de las potenciales IF-F en este estudio se requieren 406 pacientes, de acuerdo al cálculo del tamaño de muestra que se describe a continuación[16]:

$$n=\frac{Z\alpha^2 P\,(1-P)}{i^2}$$

Sustituyendo:

n= $(1.645)^2(0.40)(1-0.40)/(0.04)^2$=**406 pacientes**

n= Número de sujetos necesarios
Zα= Valor Z de tablas con α=0.05 y prueba unilateral= 1.645
P= Valor de la proporción que se supone existe en la población=0.40
i= Precisión=0.04

Para el cálculo de tamaño de muestra de factores asociados se utilizó el programaG*Power Data Analysis[17]el cual permite realizar estimaciones para regresiones con datos de conteo. Se utilizaron los valores de OR publicados por Dai D., et al.[10]; como se muestra a continuación:

	OR (IC 95%)	Estimación de n
**No. de fármacos administrados**	37.42 (34.35-40.77)	2
**Días de estancia**		
15-30	17.30 (14.16-21.13)	47
> 30	36.97 (28.11-48.61)	2

Muestreo: El tipo de muestreo fue no probabilístico por conveniencia. Las variables dependientes del estudio **HIM/2011/037** semidieron en los pacientes que ingresaron al DptoUrg durante tres etapas: Pre Intervención, Intervención y Post Intervención. Para identificar los expedientes de los pacientes que ingresaron al DptoUrg durante las etapas de Pre y Post Intervención, se solicitó al Departamento de Bioestadística y Archivo Clínico (DptoArchi) una base de datos de Excel con los números de registro, nombres, fecha de ingreso, fecha de egreso, fecha de nacimiento, sexo, edad y condición al egreso de los pacientes. Posteriormente, la lista se entregó al personal quien proporcionaba los expedientes clínicos de acuerdo a la disponibilidad de los mismos para la revisión y captura de la información en el mismo DptoArchi. Para identificar los expedientes de los pacientes que ingresaron al DptoUrg durante la etapa de Intervención, se revisaban diariamente los censos que hacían los residentes de la especialidad de Pediatría y en los que consignaban la información del número de registro, nombre, edad, sexo, diagnósticos y los días de estancia. Después, la información se capturaba y revisaba en el mismo DptoUrg de acuerdo a la disponibilidad de los mismos, dando prioridad a los pacientes que tenían mayor posibilidad de tener una RAM.

Definición de variables

Variables dependientes

Nombre	
Número de potenciales IF-F	**Definición conceptual:** Cuando la respuesta farmacológica o clínica a la administración de una combinación de medicamentos es diferente de la que se espera por los efectos conocidos de los dos medicamentos prescritos de manera individual[1]<br>**Definición operacional:** Cada lista de medicamentos de cada paciente se analizará con Medscape Drug Interaction Checker[18]. Cuando un medicamento contenga 2 o más compuestos farmacológicos (por ejemplo:Trimetoprim-sulfametoxazol) el programa los considerará de manera individual en el análisis de las potenciales IF-F. Todas las potenciales IF-F serán además categorizadas de acuerdo a su severidad en "contraindicada, grave, significante y menor". También se proporcionará una descripción de la combinación de medicamentos con potenciales IF-F, así como alguna recomendación.<br>**Tipo de variable:** Discreta<br>**Unidades de medición:**Sin unidades

Covariables

Nombre	
Edad de los pacientes	**Definición conceptual:** Tiempo transcurrido desde el nacimiento hasta la fecha de su ingreso al DptoUrg<br>**Definición operacional:** Se registró la edad consignada en la nota inicial del expediente del internamiento en el DptoUrg. Se recomiendan los siguientes puntos de corte de acuerdo a la International Conference on Harmonisation Guideline on Clinical Investigation of Medicinal Products in the Paediatric Population[19]:<br>- Infantes ≤ 23 meses<br>- Niños 24 a 143 meses (11 años 11 meses)<br>- Adolescentes ≥ 144 meses (12 años)<br>**Tipo de variable:** Nominal<br>**Unidades de medición:** Meses
Sexo de los pacientes	**Definición conceptual:** Condición orgánica que distingue al hombre de la mujer<br>**Definición operacional:** Se registró el sexo consignado en la nota inicial del expediente del internamiento en el DptoUrg<br>**Tipo de variable:** Nominal dicotómica<br>**Unidades de medición:** Hombre o mujer
Peso de los pacientes	**Definición conceptual:** Es una medida de la masa corporal total<br>**Definición operacional:** Se registró el peso consignado en la nota inicial del expediente del internamiento en el DptoUrg

	**Tipo de variable:** Continua<br>**Unidades de medición:** Kilogramos
Talla de los pacientes	**Definición conceptual:** Es la distancia máxima entre la región plantar y el vertex en un plano sagital<br>**Definición operacional:** Se registró la talla consignada en la nota inicial del expediente del internamiento en el DptoUrg<br>**Tipo de variable:** Continua<br>**Unidades de medición:** Metros
Puntaje Z de IMC de los pacientes	**Definición conceptual:** Es el número de desviaciones estándar que un valor de IMC está arriba o debajo de la media<br>**Definición operacional:** Se calculó con el programa WHO AnthroPlus. Se recomiendan los siguientes puntos de corte de acuerdo a la OMS:<br>Para niños de 0 a 5 años 11 meses:<br>- Obesidad: puntaje Z de IMC ≥ 3**(≥ 3)**<br>- Sobrepeso: puntaje Z de IMC ≥ 2 a < 3 **(2 a 2.99)**<br>- Posible riesgo de sobrepeso: puntaje Z de IMC ≥ 1 a < 2 **(1 a 1.99)**<br>- Normal: ≥ -1 a < 1 **(-1 a 0.99)**<br>- Desnutrición: < -1 **(< -0.99)**<br>Para niños de 6 a 18 años:<br>- Obesidad: puntaje Z de IMC ≥ 2**(≥ 2)**<br>- Sobrepeso: puntaje Z de IMC ≥ +1 a <+2**(1 a 1.99)**<br>- Normal: puntaje Z de IMC ≥ -1 a < +1**(-1 a 0.99)**<br>- Desnutrición: < -1 **(< -0.99)**<br>**Tipo de variable:** Ordinal<br>**Unidades de medición:** kg/m^2
Número de medicamentos	**Definición conceptual:** Número de medicamentos que se administraron a los pacientes durante su estancia en el DptoUrg<br>**Definición operacional:** Con las hojas de indicaciones de enfermería se contabilizaron los medicamentos que el paciente recibió durante su estancia en el DptoUrg<br>**Tipo de variable:** Discreta<br>**Unidades de medición:** Sin unidades
Diagnósticos	**Definición conceptual:** Es el procedimiento por el cual se identifica una enfermedad, entidad nosológica, síndrome o cualquier estado patológico o de salud<br>**Definición operacional:** Se registraron los diagnósticos de base de todos los pacientes consignados en la nota inicial del expediente del internamiento y se clasificaron de acuerdo a la CIE 10<br>**Tipo de variable:** Nominal<br>**Unidades de medición:** Sin unidades
Condición al egreso	**Definición conceptual:** Estado de salud en el que egresa el paciente del DptoUrg<br>**Definición operacional:** El DptoArchi proporcionó una base de datos de cada uno de los pacientes que categoriza a la variable en: alta voluntaria, curado, mejorado, traslado y

	defunción<br>**Tipo de variable:**Nominal<br>**Unidades de medición:**Sin unidades
Presencia de RAMs	**Definición conceptual:** Una respuesta la cual es nociva y no intencionada y ocurre a dosis normalmente usadas en humanos para la profilaxis, diagnóstico, tratamiento o para la modificación de una función fisiológica[20]<br>**Definición operacional:** En todos pacientes se evaluaron las RAMs con el Algoritmo de Naranjo<br>**Tipo de variable:**Nominal dicotómica<br>**Unidades de medición:**Si o No

Análisis estadístico

Para la captura de la información se diseñó una base de datos en Microsoft Office Excel 2007 la cual posteriormente se exportó a SPSS versión 18 y STATA versión 13para realizar los análisis estadísticos.

Calidad de la información: Se exportaron a SPSS versión 18 las variables: número de expediente, nombre del paciente, fecha de nacimiento, edad, sexo, peso, talla, diagnósticos de ingreso, etapa del estudio, fecha de ingreso, presencia de RAM, fecha de egreso y medicamentos prescritos durante su estancia (nombre, dosis, vía de administración y fechas). Para cada paciente se calcularon las variables: Z de IMC, y número de medicamentos administrados durante su estancia. Posteriormente, se verificaron los valores extremos (mínimos y máximos). Se ingresaron a la base de datos 1,179 pacientes, en los cuales se identificó que 120 tenían al menos un faltante de peso, edad y talla. Los pacientes que tenían faltantes sólo en la variable de peso fueron 71, sólo en la variable de talla 119, en la variable de edad 44, en la variable de sexo ninguno y en la variable del número de medicamentos administrados durante su estancia 101. La imputación de los datos se realizó con regresiones y tablas de la OMS de peso y talla para la edad en niños y niñas, de acuerdo a las combinaciones que se describen a continuación. Con regresiones se asignaron 47 valores a la variable talla y solamente 1 a la variable edad. Usando las tablas de la OMS de peso y talla para la edad en niños y niñas se asignaron 29 valores a las variables de peso y talla. Para finalizar, se identificaron 61 casos que no tenían la información correspondiente al número de medicamentos pero que si tenían la relacionada al sexo, peso, edad y talla. A estos casos se les asignó la mediana del número de medicamentos administrados durante su estancia, porque la distribución de la variable no fue normal. En los análisis del estudio **HIM/2011/037**se

incluyeron 1,136 pacientes de los cuales 915 cumplen los criterios de inclusión para este análisis.

Análisis descriptivo y bivariado: El análisis descriptivo se realizará con medidas de tendencia central y de dispersión como el número de casos (%), media (IC 95%) y mediana (percentiles 25-75) de acuerdo a la naturaleza de cada variable (cuantitativa o cualitativa). Para el análisis bivariado las variables de edad, sexo, talla, peso, Z de IMC y número de medicamentos se contrastaran entre el grupo de pacientes que presentaron interacción y los que no presentaron interacción. Se realizarán las pruebas de normalidad (Kolmogorov-Smirnov y Shapiro-Wilk) para analizar la distribución de las variables talla, peso y número de medicamentos y poder elegir entre las pruebas paramétricas (t de Sudent) o no paramétricas (U de Mann-Whitney). Para las variables categóricas de grupo de edad, sexo, Z de IMC, diagnósticos, condición al egreso y presencia de RAMs se realizará una prueba de Chi cuadrada. Se considerará $p < 0.05$ como estadísticamente significativa. Los análisis se realizarán con SPSS versión 18y se considerará $p < 0.05$ como estadísticamente significativa.

Análisis multivariado: Se realizarán Regresiones para Datos de Conteo (Regresión Binomial Negativa, Regresión Binomial Negativa con Ceros Inflados, Regresión de Poisson). Para elegir el modelo con el que se obtiene el mejor ajuste se utilizarán los criterios de información de Akaike y Bayesiano.Una vez elegido el modelo que mejor ajuste se estimarán los coeficientes de regresión, junto con el comando 'listcoef', que estima el cambio porcentual en el valor esperado de la variable de conteo. Los análisis se realizarán con el programa STATA versión 13y se considerará $p < 0.05$ como estadísticamente significativa.

Procedimientos

Captura de la información de los pacientes: Como se mencionó en la sección de Muestreo las variables dependientes del estudio **HIM/2011/037** semidieron en los pacientes que ingresaron al DptoUrg durante tres etapas (Pre Intervención, Intervención y Post Intervención) debido a que se trataba de un estudio de intervención. Para identificar los expedientes de los pacientes que ingresaron al DptoUrg durante las etapas de Pre y Post Intervención, se solicitó al Departamento de Bioestadística y Archivo Clínico (DptoArchi) una base de datos de Excel con los números de registro, nombres, fecha de ingreso, fecha de egreso, fecha de nacimiento, sexo, edad y condición al egreso de los pacientes. Posteriormente, la lista se entregó al personal quien proporcionaba los expedientes clínicos de acuerdo a la disponibilidad de los mismos para la revisión y captura de la información en el mismo DptoArchi. La información que

se capturó en Microsoft Office Excel 2007 fueron los aspectos demográficos de los pacientes (número de expediente, nombre, fecha de nacimiento, edad, sexo, peso, talla), fecha de ingreso, diagnósticos de ingreso, resultados de laboratorio, datos relevantes de la historia clínica y de los medicamentos administrados durante su ingreso (nombre, dosis, vía de administración, indicaciones, fechas de inicio y término) y fecha de egreso del DptoUrg. Durante la revisión del expediente clínico se verificó si el paciente había presentado una RAM que fue valorada con el algoritmo de Naranjo. Para identificar los expedientes de los pacientes que ingresaron al DptoUrg durante la etapa de Intervención, se revisaban diariamente los censos que hacían los residentes de la especialidad de Pediatría y en los que consignaban la información del número de registro, nombre, edad, sexo, diagnósticos y los días de estancia. La información que se capturó durante esta etapa fue la misma que en las etapas de Pre y Post Intervención sólo que esta actividad se realizó en el mismo DptoUrg de acuerdo a la disponibilidad de los expedientes y dando prioridad a los pacientes que tenían mayor posibilidad de tener una RAM.

Búsqueda de las potenciales interacciones fármaco-fármaco: Para la evaluación e identificación de las interacciones se utilizará el programa de interacciones Medscape Drug Interaction Checker[18]. El análisis se realizará ingresando al programa los medicamentos prescritos de cada uno de los pacientes. Se seleccionó esta base de datos porque estratifica el nivel de significancia de la interacción en:contraindicada, grave, significante y menor (tabla 1), y porque explica el mecanismo de cada interacción y provee recomendaciones de manejo clínico de la potencial interacción. Cuando un medicamento contenga 2 o más compuestos farmacológicos (por ejemplo Trimetoprim-sulfametoxazol) el programa los considera de manera individual en el análisis de las potenciales IF-F.

Tabla 1.- Nivel de severidad de una potencial IF-F de acuerdo a MedscapeDrugInteractionChecker

**Nivel de severidad**	**Descripción**
Contraindicada	Nunca se use esta combinación de medicamentos debido al alto riesgo de una interacción peligrosa
Grave	Potencialmente se puede presentar una interacción grave, se requiere la vigilancia regular del médico o que se modifique el medicamento
Significante	Potencialmente se puede presentar una interacción significante, probablemente se requiera la vigilancia del médico
Menor	Es improbable y no significativa

Consideraciones Éticas y de Bioseguridad

El protocolo principal titulado "Estudio comparativo de las reacciones adversas a medicamentos entre la Farmacovigilancia Intensiva y la Notificación Espontánea en un Departamento de Urgencias de un hospital pediátrico" con número **HIM/2011/037**fue aprobado por las Comisiones de Investigación, Ética y Bioseguridad del Hospital Infantil de México Federico Gómez el día 3 de mayo de 2012**(Anexo 1),** con lo relacionado a este protocolo cabe hacer notar las siguientes consideraciones:

Ética: El protocolo corresponde a "Investigación sin riesgo" ya que es un método de investigación documental, en el que no se realiza intervención o modificación relacionada con variables fisiológicas, psicológicas o sociales, y corresponde a una revisión de una base de datos **(Formato Adjunto)**

Bioseguridad: De acuerdo a las instrucciones de llenado del "Formato de Bioseguridad" consideramos que este protocolo "No tiene implicaciones de Bioseguridad"**(Formato Adjunto)**

X. LIMITACIONES DEL ESTUDIO

Las limitaciones del estudio que deben ser consideradas en la interpretación de los resultados son:

- El estudio no se centrará en las consecuencias clínicas de las interacciones medicamentosas, sino en su potencial aparición
- El estudio fue conducido solamente en un departamento de un hospital y probablemente al extrapolar los resultados a otros hospitales o departamentos se encuentren variaciones debido a las diferencias en la población, las especialidades y al tipo de medicamentos empleados

XI. BIBLIOGRAFÍA

1 Mino-León D, Galván-Plata ME, Doubova SV, Flores-Hernandez S, Reyes-Morales H. A pharmacoepidemiological study of potential drug interactions and their determinant factors in hospitalized patients. Rev Invest Clin. 2011;63(2):170-8

2 Ganeva M, Gancheva T, Troeva J, Kiriyak N, Hristakieva E. Clinical relevance of drug-drug interactions in hospitalized dermatology patients. AdvClinExp Med. 2013;22(4):555-63

3 Dechanont S, Maphanta S, Butthum B, Kongkaew C. Hospital admissions/visits associated with drug-drug interactions: a systematic review and meta-analysis. Pharmacoepidemiol Drug Saf. 2014; 23(5):489-97

4 Pirmohamed M, James S, Meakin S, Green C, Scott AK, Walley TJ, Farrar K, Park BK, Breckenridge AM. Adverse drug reactions as cause of admission to hospital: prospective analysis of 18 820 patients. BMJ. 2004;329(7456):15-9

5 Kearns GL, Abdel-Rahman SM, Alander SW, Blowey DL, Leeder JS, Kauffman 230 RE. Developmental pharmacology--drug disposition, action, and therapy in infants and children. N Engl J Med. 2003;349(12):1157-1167

6 Lindell-Osuagwu L, Korhonen MJ, Saano S, Helin-Tanninen M, Naaranlahti T, Kokki H. Off-label and unlicensed drug prescribing in three paediatric wards in Finland and review of the international literature. J Clin Pharm Ther. 2009; 34(3):277-287

7 Feudtner C, Dai D, Hexem KR, Luan X, Metjian TA. Prevalence of polypharmacy exposure among hospitalized children in the United States. Arch PediatrAdolesc Med. 2012 Jan;166(1):9-16

8 Feinstein J, Dai D, Zhong W, Freedman J, Feudtner C. Potential drug-drug interactions in infant, child, and adolescent patients in children's hospitals. Pediatrics. 2015; 135(1):e99-108

9 Oshikoya KA, Oreagba IA, Ogunleye OO, Lawal S, Senbanjo IO. Clinically significant interactions between antiretroviral and co-prescribed drugs for HIV-infected children: profiling and comparison of two drug databases. TherClin Risk Manag. 2013; 9:215-21

10 Dai D, Feinstein JA, Morrison W, Zuppa AF, Feudtner C. Epidemiology of Polypharmacy and Potential Drug-Drug Interactions Among Pediatric Patients in ICUs of U.S. Children's Hospitals. PediatrCrit Care Med. 2016; 17(5):e218-28

11 Fernández de Palencia Espinosa MA, Díaz Carrasco MS, Fuster Soler JL, Ruíz Merino G, De la Rubia Nieto MA, Espuny Miró A. Pharmacoepidemiologicalstudy of drug-

druginteractions in onco-hematologicalpediatricpatients. Int J Clin Pharm. 2014; 36(6):1160-9
12 Langerová P, Prokeš M, Konvalinka M, Fürstová J, Urbánek K. Incidence of potential drug interactions in medication prescriptions for children and adolescents in the University Hospital Olomouc, Czech Republic. Eur J Pediatr. 2013;172(5):631-8
13 Morales Ríos O, Jasso Gutiérrez L, Talavera JO, Téllez-Rojo MM, Olivar López V, Garduño Espinosa J, Muñoz Hernández O. A comprehensive intervention for adverse drug reactions identification and reporting in a Pediatric Emergency Department. Int J Clin Pharm. 2016;38(1):80-7
14 DoubovaDubova SV, Reyes-Morales H, Torres-Arreola L del P, Suárez-Ortega M. Potential drug-drug and drug-disease interactions in prescriptions for ambulatory patients over 50 years of age in family medicine clinics in Mexico City. BMC Health Serv Res. 2007;7:147
15 Cook TD, Campbell DT, eds. Quasi-Experimentation. Design and analysis issues for field settings. Chicago: Rand McNally College Publising Company; 1979
16 Martínez-González MA, eds. Bioestadística Amigable. España: Diaz de Santos; 2006
17 G*Power Data Analysis. http://www.ats.ucla.edu/stat/gpower/indepsamps.htm. Accesed 17 September 2016
18 Drug Interaction Checker. http://reference.medscape.com/drug-interactionchecker. Accesed 17 May 2016
19 European Medicines Agency. ICH Topic E 11. Clinical Investigation of Medicinal Products in the Paediatric Population. http://www.ema.europa.eu/docs/en_GB/document_library/Scientific_guideline/2009/09/WC500002926.pdf. Accesed 17 September 2015
20 Uppsala Monitoring Centre. http://www.who-umc.org/. Accesed 17 September 2015

XII. ANEXO 1

HOSPITAL INFANTIL DE MÉXICO FEDERICO GÓMEZ

SALUD
SECRETARÍA DE SALUD

Dirección General

México, D.F., 3 de mayo de 2012 1000/ 468 /2012

Dr. Luis Jasso Gutiérrez
Jefe del Departamento de Evaluación y
Análisis de Medicamentos
Presente

Informo a usted que las Comisiones de Investigación, Ética y Bioseguridad, después de haber revisado su protocolo número **HIM/2011/037** " Estudio comparativo de las reacciones adversas a medicamentos entre la Farmacovigilancia Intensiva y la Notificación Espontánea en un Departamento de Urgencias de un hospital pediátrico", han emitido el dictamen de:

APROBADO

en los términos y condiciones señalados por dichas Comisiones. Por lo anterior, se autoriza su desarrollo.

Atentamente

Dr. José Alberto García Aranda
Director General

Con copia:
C.P. Elías Hernández Ramírez. Jefe de la Unidad Auxiliar Administrativa de Proyectos de Investigación.

OMH/glro

INSTITUCION DE SERVICIO MEDICO, ENSEÑANZA E INVESTIGACION, AFILIADA A LA UNAM
Dirección: Dr. Márquez 162, Col. Doctores, Delegación Cuauhtémoc, 06720, México, D.F.
Tel: 57 61 01 81/ 57 61 19 63/ 57 61 36 60; Fax: 57 61 89 74; Conm. 52 28 99 17
www.himfg.edu.mx
