# Supplementary material for: Potential drug-drug interactions and their risk factors in pediatric patients admitted to the emergency department of a tertiary care hospital in Mexico
Source: PLoS One. 2018 Jan 5;13(1):e0190882. doi: 10.1371/journal.pone.0190882 (PMC5755936; doi:10.1371/journal.pone.0190882)
Supplement: S2 File — (PDF) [file pone.0190882.s006.pdf]

México, D.F., a 7 de Diciembre de 2016

DG/1000/ **1087** /2016

Dra. Olga Magdala Morales Ríos
Departamento de Evaluación y Análisis de Medicamentos
Presente

Informo a usted, que los Comités de Investigación, Ética y Bioseguridad, después de haber revisado su protocolo **HIM 2017-008** "Estudio fármaco epidemiológico de potenciales interacciones fármaco-fármaco en niños hospitalizados en un Departamento de Urgencias de un Hospital Pediátrico de tercer nivel en México", han emitido el dictamen de:

APROBADO

En los términos y condiciones señalados por dichos Comités. Por lo anterior, se autoriza su desarrollo.

Atentamente

Dr. José Alberto García Aranda
Director General

Con copia:
Lic. Martha Reynoso Robles. Jefa del Departamento Auxiliar Administrativo.

JAGA/JGE/JGO/ash
